# A Review of Neurogenic Stunned Myocardium

**DOI:** 10.1155/2017/5842182

**Published:** 2017-08-10

**Authors:** Sylvia Biso, Supakanya Wongrakpanich, Akanksha Agrawal, Sujani Yadlapati, Marina Kishlyansky, Vincent Figueredo

**Affiliations:** ^1^Department of Internal Medicine, Einstein Medical Center, Philadelphia, PA, USA; ^2^Einstein Institute for Heart and Vascular Health, Einstein Medical Center, Philadelphia, PA, USA; ^3^Sidney Kimmel Medical College at Thomas Jefferson University, Philadelphia, PA, USA

## Abstract

Neurologic stunned myocardium (NSM) is a phenomenon where neurologic events give rise to cardiac abnormalities. Neurologic events like stroke and seizures cause sympathetic storm and autonomic dysregulation that result in myocardial injury. The clinical presentation can involve troponin elevation, left ventricular dysfunction, and ECG changes. These findings are similar to Takotsubo cardiomyopathy and acute coronary syndrome. It is difficult to distinguish NSM from acute coronary syndrome based on clinical presentation alone. Because of this difficulty, a patient with NSM who is at high risk for coronary heart disease may undergo cardiac catheterization to rule out coronary artery disease. The objective of this review of literature is to enhance physician's awareness of NSM and its features to help tailor management according to the patient's clinical profile.

## 1. Introduction

Every year, an estimated 800,000 people in the United States have a new or recurrent stroke. Roughly every 40 seconds, a person experiences a stroke, and every 4 minutes, a person dies from one [1]. Stroke is a significant cause of major long-term functional disability [2]. Cardiovascular complications are common after an acute stroke [3]. Neurologic events have been known to cause cardiac abnormalities ranging from arrhythmias, ventricular dysfunction, and myocardial infarction to sudden cardiac death [4]. In a study by Chin et al., approximately 13% of patients had an associated acute myocardial infarction within 3 days of a cerebrovascular accident [5]. During the first month after a stroke, cardiac events account for the most common nonneurologic cause of mortality [6]. In addition, the most common cause of death on long-term follow-up after a cerebrovascular accident is annual cardiac mortality [7]. Even 3 to 4 years after a stroke, there is still a 2% annual risk of myocardial infarction [8].

Neurogenic myocardial injury, such as neurogenic stunned myocardium (NSM), is a phenomenon where cardiac complications occur after a neurologic event (i.e., stroke, subarachnoid hemorrhage, or seizures) due to dysregulation of the autonomic nervous system [9]. It can manifest as almost any cardiac disturbance [10]. NSM oftentimes can be very similar to myocardial infarction, presenting with ischemic ECG changes, decreased cardiac function, elevated troponin levels, and ventricular wall abnormalities [11, 12]. A surge of catecholamines after areas of the brain governing the autonomic system have been damaged has been suggested as a potential mechanism [13]. Unlike myocardial infarction, where coronary arteries are blocked, there is no significant obstruction of the coronary vessels in patients with an NSM causing the myocardial infarction-like signs and symptoms. A study by Kono et al. on subarachnoid hemorrhage patients who had ST-segment elevation and corresponding wall motion abnormalities on echocardiogram revealed no coronary artery stenoses or vasospasm of epicardial arteries on coronary angiography [14].

NSM after acute ischemic stroke or subarachnoid hemorrhage is similar to Takotsubo cardiomyopathy in presentation. They both mimic myocardial infarction and have transient left ventricular hypokinesis [15]. Takotsubo cardiomyopathy usually presents with a regional wall motion abnormality, while NSM shows global hypokinesis. Due to their catecholamine-mediated natures, both are thought to have the same pathophysiology and follow the same course of illness [16]. In terms of treatment, both are reversible and are medically managed [17]. Some believe that they are the same entity and that they should both fall under stress-induced cardiomyopathy [18, 19].

Distinguishing myocardial injury caused solely by a neurologic event from an acute myocardial infarction is difficult. For patients with subarachnoid hemorrhage, Bulsara et al. [20] recommended the following guidelines for distinguishing NSM from acute myocardial infarction: (1) no history of heart disease, (2) new onset of cardiac dysfunction (ejection fraction < 40%), (3) wall motion abnormalities that do not correspond to ischemic ECG changes, and (4) troponin values less than 2.8 ng/ml in patients with EF < 40%. Unlike subarachnoid hemorrhage patients, however, acute ischemic stroke patients commonly have preexisting heart disease and extensive atherosclerotic disease [21]. These criteria do not apply to them. The objective of this review of literature, thus, is to enhance physician's awareness of NSM and its features to help in the differential diagnosis and assist in tailoring the management according to the patient's clinical profile.

## 2. Neuroanatomic Correlates 

Studies have demonstrated that involvement of the insular cortex during stroke results in autonomic dysregulation and adverse cardiac outcomes [22]. The insular cortex, considered the “hidden fifth lobe,” is situated at the base of the Sylvian fissure. Grossly, it consists of the anterior and posterior insula, bisected by the central insular sulcus [23]. The insula integrates autonomic, motor, and sensory functions through its reciprocal connections with other parts of the brain, notably the limbic system [24]. It is particularly important for gustatory sensation, articulation planning and execution, vestibular function, and control of the autonomic nervous system [25]. Its importance in the pathophysiology of NSM lies in its ability to maintain the balance between the sympathetic and parasympathetic system.

The insula has a major role in cardiovascular regulation, specifically in limbic-autonomic regulation [26]. The insular cortex contains baroreceptive units of sympathoexcitatory and sympathoinhibitory neurons that regulate blood pressure and heart rate. Stimulation or injury of the insula gives rise to changes in these cardiovascular parameters. Lesions in this part of the brain have been found to result in an increase in norepinephrine levels in the blood which cause left ventricular dysfunction [27]. Stimulation of the right insula elicited sympathetic effects like tachycardia and hypertension, while stimulation of the left insula resulted in parasympathetic responses such as bradycardia and vasodepressor effects [28]. A left insular stroke has been shown to cause depressed parasympathetic regulation and increased sympathetic tone. The reverse is true for right insular stroke [7]. Both right and left insular lesions may also result in decreased heart rate variability. Although data vary regarding which side is more associated with adverse cardiac outcomes, both have been associated with increased cardiac morbidity and mortality [29].

The parietal lobe has also been associated with NSM but its role is less clear. The parietal lobe and the insula are supplied by the same blood vessel, the middle cerebral artery. Because of this, when the insula suffers an ischemic insult, the parietal lobe is also affected. It has been postulated that the insula is the one causing the myocardial injury and the parietal lobe happens to be a “bystander” [25]. A large study by Rincon et al., however, did not support these findings [30]. Its results showed that the parietal lobe stroke was an independent predictor for cardiac death or myocardial infarction. By contrast, insular cortex lesions had no correlation to cardiac events. However, in this study, only 40% of the patients had an MRI. The study relied heavily on CT scans that could have led to misclassification of the stroke lesions. Another limitation of the study was that the study was not primarily designed to find the neuroanatomic correlates of NSM. The results obtained were only a secondary analysis of their data. Thus, more research is needed to clarify the extent of the influence of the parietal lobe on NSM.

## 3. Pathophysiology

**Figure 1:**
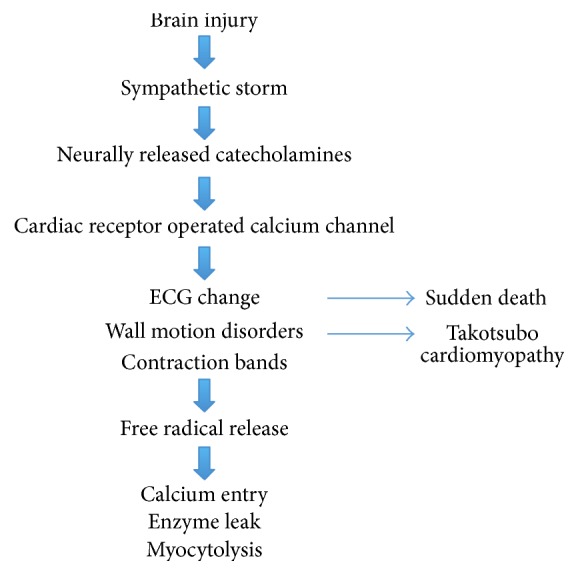
Pathophysiology of NSM.

Excessive sympathetic stimulation is the underlying mechanism for neurogenic myocardial injury (Figure 1). The cascade of events starts with an influx of catecholamines from a neurologic lesion or extreme stress. Cardiac injury then happens in three possible ways: (1) coronary vasospasm secondary to the catecholamine surge [11], (2) ischemia via increase in myocardial demand [9], and (3) direct toxic effects of catecholamines on the myocardium [31]. Toxic effects of catecholamines can occur via calcium overload of the cardiomyocytes [32]. Excessive stimulation of the B-adrenergic receptors by catecholamines opens calcium channels. The abnormal amount of calcium into the cells causes contractile dysfunction and adenosine triphosphate (ATP) depletion. ATP depletion then leads to mitochondrial dysfunction culminating in cell death [9].

The resulting histopathology of NSM is different from those found in patients with myocardial infarction. In patients with coronary artery disease, histopathology shows myocardial necrosis in the distribution of the coronary arteries [13]. Neurogenic cardiac injury, in contrast, is predominantly myofibrillar degeneration or myocytolysis that is transient and distinct from myocardial necrosis. Myocytolysis or myofibrillar degeneration denotes acute myocardial stress and is characterized by foci of subendocardial hemorrhage surrounding epicardiac nerves [33]. Because of the myocytolysis' subendocardial location where the cardiac conducting system is also present, arrhythmias are common in NSM [24].

## 4. Electrocardiographic Features 

**Figure 2:**
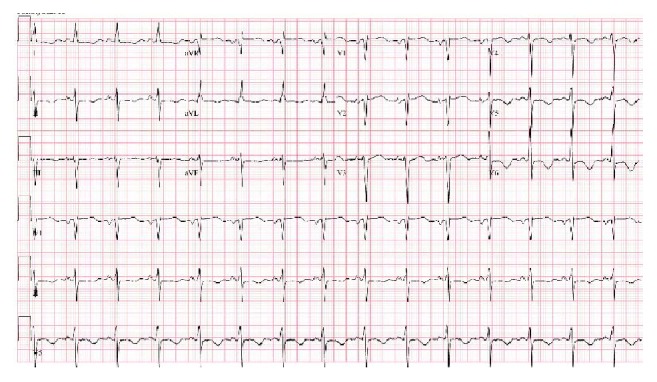
New T-wave inversions in inferolateral leads in a patient with NSM.

Abnormal ECG findings can be observed in 60%–90% of cerebral infarction patients (Figure 2) [34]. The most common morphologic change is a prolonged QT interval. This is followed by T-wave inversions and then ST-depression [35]. The most common rhythm change is atrial fibrillation followed by sinus tachycardia [36]. A triad of ECG findings, QT prolongation and U waves and T waves' inversion, which are associated with acute neurologic lesions, were seen in 8% of patients after a cerebral infarction [35].

A study by Dogan and colleagues observed ischemic ECG changes in 90% of ischemic and hemorrhagic stroke patients regardless of whether they have history of cardiac disease or not [37]. The most common ECG changes were nonspecific ST-T wave changes, followed by ST-segment depression [38, 39]. A study by McDermott et al. found that ST-segment change is an independent predictor of early death and is the most important long-term prognostic factor apart from age [40, 41]. In Dogan et al.'s study, ischemic ECG changes were not associated with mortality, but history of cardiac disease was found to be an independent predictor of mortality [37]. Others have attempted to map ECG abnormalities to specific brain locations; however, no strong associations have been found [42].

Arrhythmias are common after stroke and they occur more frequently in patients with cardiac disease. Other predictors for cardiac arrhythmias after stroke are older age, hypertension, and diabetes mellitus [43]. The National Institutes of Health Stroke Scale (NIHSS) score is an independent predictor for the appearance of arrhythmias. Thus, the NIHSS score on admission could be used to stratify patients and find out who needs cardiac monitoring after 24 hours. Arrhythmias found in acute stroke in the order of frequency are atrial fibrillation, sinus tachycardia, premature ventricular complexes, and ventricular tachycardia [44, 45].

## 5. 2D Echocardiographic Features

NSM can present with left ventricular dysfunction [18]. A study by Banki et al. in subarachnoid hemorrhage patients with cardiac dysfunction observed that the patients' wall motion abnormalities on echocardiograms matched their ECG changes [19]. However, in another study by Bulsara, involving acute ischemic cerebral infarct patients, echocardiograms did not match their ECG findings. Average ejection fraction was found to be 33%. After 5 days, cardiac output improved by 1.6 L/minute [20]. In patients with subarachnoid hemorrhage, reversal of wall motion abnormalities was observed in 2 days [9].

## 6. Troponin Levels

NSM can be asymptomatic and underdiagnosed in stroke patients. Elevated troponin levels in patients with ischemic stroke can occur without corresponding chest pain, ECG changes, or echocardiographic changes. It can be the only sign of cardiac injury. In a study by Faiz and colleagues, elevated troponin T levels occurred in 53% of acute ischemic stroke patients. Only 6% of patients, however, met the criteria for MI [46]. In another study by Jensen et al. of patients without overt ischemic heart disease, elevated troponin levels occurred only in 3% [47]. Presence of congestive heart failure or renal failure can account for the increase in troponin levels in these patients.

There are many factors that are independently related to increased troponins in stroke patients. These include age, previous coronary artery disease, congestive heart failure, diabetes, hypercholesterolemia, and chronic kidney disease. Stroke patients with involvement of the insula are more commonly found to have increased troponin levels. When patients with chronic kidney disease and coronary artery disease were excluded, only insular involvement was found to be significantly related to elevated troponin levels [48, 49].

Elevated troponin T levels were also found to predict poor outcome in patients with acute stroke. In a study by Ghali et al., troponin was found to have sensitivity of 0.27, specificity of 0.94, and likelihood ratio (LR) of 4.5 for predicting a poor outcome after ischemic stroke [50, 51]. Troponin was also correlated to the severity of stroke [50]. However, NIHSS has been proven to be a stronger predictor of adverse outcomes. It has stronger sensitivity (0.78), specificity (0.96), and LR (17.7) compared to troponin T [51]. Another study by Jensen et al. found that patients with increased troponin levels were at increased risk of death in the hospital and within the following 2 years [47, 50]. Studies regarding troponin levels and acute ischemic stroke are abundant but limited by the heterogeneity of laboratory assay, different laboratory references, and cut-offs for what constitutes a significant elevation [52, 53].

## 7. Management

Silent coronary artery disease has been found to be prevalent in patients with acute ischemic stroke [54]. Coronary heart disease and ischemic stroke have the same risk factors and they tend to occur in the same subset of patients [55]. Given the concurrence of acute ischemic stroke and cardiac abnormalities, evaluation for myocardial dysfunction and injury is important in these patients. This is especially true as cardiac abnormalities that can occur are often asymptomatic, possibly because of the decreased cognitive and functional capacities of stroke patients. Furthermore, cardiac events are also a major cause of mortality and morbidity in these patients [6].

An American Heart Association/American Stroke Association (AHA/ASA) statement recommends that patients with ischemic stroke or TIA undergo cardiovascular risk assessment and aggressive risk reduction management [56]. They further recommend that patients with multiple coronary artery disease risk factors or high Framingham risk score (i.e., 10-year coronary heart disease risk >/= 20%) should be considered for noninvasive coronary artery disease testing. ACC/AHA practice guidelines, in addition, can help determine which diagnostic test for coronary heart disease can be used [57]. Finally, the decision to institute medical, endovascular, or surgical management must be tailored according to patient's clinical profile.

On the other hand, if the cardiac abnormality is likely from NSM, such as in subarachnoid hemorrhage patients, coronary angiography is not routinely recommended. In a study by Kono and colleagues involving subarachnoid patients with ST-segment elevation [14], coronary angiography revealed no stenosis or vasospasm of coronary arteries. However, a strong clinical suspicion of plaque rupture or high burden of atherosclerosis may still warrant coronary angiography.

## 8. Conclusion

Cardiovascular complications in the setting of neurologic events are diverse in presentation and can produce adverse outcomes. The pathophysiology involves excessive catecholamine release during a neurologic injury that produces increased cardiac demand, stress, and myocytolysis. Most studies showed that the insular cortex is mostly involved in causing this autonomic dysregulation, especially in stroke patients. The resulting clinical manifestations are varied and can mimic acute coronary syndromes. It is difficult, if not impossible, to distinguish NSM from acute coronary syndrome based on clinical manifestations alone. There are also no clear guidelines on how to manage NSM. In patients with risk factors for coronary heart disease, cardiovascular risk assessment, aggressive cardiovascular risk reduction, and tailoring the management into medical, endovascular, or surgical management depending on the clinical characteristics of the patient are currently employed.
